# The Ovarian Sensitivity Index (OSI) Significantly Correlates with Ovarian Reserve Biomarkers, Is More Predictive of Clinical Pregnancy than the Total Number of Oocytes, and Is Consistent in Consecutive IVF Cycles

**DOI:** 10.3390/jcm9061914

**Published:** 2020-06-18

**Authors:** Alberto Revelli, Gianluca Gennarelli, Valentina Biasoni, Alessandra Chiadò, Andrea Carosso, Francesca Evangelista, Carlotta Paschero, Claudia Filippini, Chiara Benedetto

**Affiliations:** 1 Obstetrics and Gynecology 1, Physiopathology of Reproduction and IVF Unit, S. Anna Hospital, Department of Surgical Sciences, University of Torino, 10126 Torino, Italy; gennarelligl@gmail.com (G.G.); valentina_biasoni@yahoo.it (V.B.); ale85.ac@libero.it (A.C.); andrea88.carosso@gmail.com (A.C.); francesca-evangelista@hotmail.it (F.E.); carlotta.paschero@libero.it (C.P.); chiara.benedetto@unito.it (C.B.); 2 Clinical Statistics, Department of Surgical Sciences, University of Torino, 10126 Torino, Italy; claudia.filippini@unito.it

**Keywords:** controlled ovarian stimulation, in vitro fertilization, ovarian sensitivity index, ovarian responsiveness, biomarkers

## Abstract

Background and Objectives: Some biomarkers of ovarian responsiveness to gonadotropins and the total number of retrieved oocytes are known to affect the success rate after controlled ovarian stimulation (COS) and in vitro fertilization (IVF). The aim of this study was to study another putative marker, the Ovarian Sensitivity Index (OSI: (number of retrieved oocytes/total gonadotropin dose) × 1000), assessing whether (a) it correlates with ovarian responsiveness biomarkers, (b) it is an independent predictor of clinical pregnancy, (c) it predicts clinical pregnancy comparably to the number of retrieved oocytes, and (d) it is consistent in the repeated COS cycles of the same woman. Design: retrospective analysis. Setting: public IVF Unit in University Hospital. Cases and Measurements: 1612 patients submitted to 3353 IVF cycles were included, their OSI was calculated and it was correlated with the ovarian responsiveness biomarkers (age, BMI, anti-Mullerian hormone, antral follicle count). The OSI and the total number of oocytes were compared for their value in predicting clinical pregnancy. The inter-cycle consistency of the OSI was estimated in 209 patients who underwent two consecutive cycles in which the ovarian stimulation regimen was changed from the Gonadotropin-releasing Hormone (GnRH)-agonist long protocol to the GnRH-antagonist protocol or vice-versa. Results: The OSI turned out to be significantly related to age and BMI (inversely), the anti-Mullerian hormone (AMH) and the antral follicle count (AFC) (directly), to be an independent predictor of clinical pregnancy, and to correlate with clinical pregnancy better than the total number of oocytes (*p* < 0.0001 vs. <0.002). In patients who underwent two consecutive COS cycles changing stimulation regimen, the OSI showed 82% consistency. Conclusion(s): The OSI significantly correlates to the currently used biomarkers of ovarian responsiveness; it is an independent predictor of clinical pregnancy; it is more predictive of clinical pregnancy than the total number of oocytes, and is highly consistent in repeated IVF cycles even when the COS protocol changes. These characteristics make the OSI quite suitable to be incorporated into more complex prediction models of IVF outcome.

## 1. Introduction

Among the factors affecting the outcome of human in vitro fertilization (IVF), the ovarian response to the controlled ovarian stimulation (COS) is one of the most important. Studies performed on large cohorts of patients have clearly shown that the number of oocytes obtained at ovum pick-up (OPU) is a major variable, conditioning both the live birth rate (LBR) after fresh embryo transfer (ET) [1], and the cumulative LBR after transferring fresh and thawed embryos [2,3].

Since the retrieval of 15–18 oocytes was found to be associated with optimal IVF outcomes, the idea of tailoring the COS in order to reach that target figure has become very popular. In actuality, the full potential of the ovary is expressed only when a maximal gonadotropin dose is administered, but often this does not happen for safety issues, e.g., the need to avoid a life-threatening complication such as the severe form of ovarian hyperstimulation syndrome (OHSS). Hence, the need for tailoring the COS is accomplished through an individualized choice of the ovarian stimulation protocol, and of the type and dose of medications [4]. For this purpose, the clinical (e.g., age, body mass index), endocrine (e.g., anti-Mullerian hormone), and ultrasound-related (e.g., antral follicle count) biomarkers are widely used as the potential predictors of ovarian response [5]. Whereas no marker has proven so far to be reliable enough in predicting clinical pregnancy [6], the response to the COS in terms of the follicular recruitment is predictable to some extent, although not in all cases.

As a matter of fact, the ovarian sensitivity to exogenous gonadotropins (FSH, LH, hMG or their combination) may vary also according to factors intrinsic to the patient’s genetics. The polymorphic variants of the FSH-receptor [7] and the individual rhythm of follicular maturation waves [8] are examples of the complex, individual regulation of follicular recruitment and growth. The clinical consequence of the individual variability of these intrinsic factors is the occurrence of an unexpected ovarian response during the COS.

The observation that the total number of retrieved oocytes was sometimes unable to accurately reflect the ovarian potential has stimulated the research of other markers of ovarian response, such as the follicular output rate (FORT) [9] and the follicle-oocyte index (FOI) [10]. Both the FORT (ratio between the number of pre-ovulatory and the pre-stimulation follicles) and the FOI (ratio between retrieved oocytes and pre-stimulation follicles) have been claimed to better reflect the dynamic nature of follicular growth in response to exogenous gonadotropins, and have been found to be positively related to the outcome of IVF.

Another marker of the ovarian potential to produce oocytes in response to hormonal stimulation was identified in the ratio between the number of retrieved oocytes and the total administered gonadotropin dose, that was named the Ovarian Sensitivity Index (OSI) [11]. The OSI soon became an interesting tool in estimating the ovarian sensitivity to exogenous gonadotropins and was mainly used to adjust the COS regimen in subsequent IVF cycles, following the first stimulation. It may vary widely between women in comparable ranges of ovarian reserve. Indeed, one author showed that the probability of clinical pregnancy after IVF was similar with a high number of oocytes obtained after receiving a high gonadotropin dose (low OSI), and with a much lower number of oocytes obtained after a low dose stimulation (high OSI) [12].

The OSI represents an interesting tool to estimate the ovarian sensitivity and allows a tailored, individual adjustment of the COS in subsequent IVF cycles. Although introduced in clinical practice some years ago, the reliability of the OSI as a marker of IVF-induced clinical pregnancy has never been thoroughly studied.

In the present study, we aimed to: (a) better clarify the relationship between the OSI and the other OR markers, such as the age, basal antral follicle count (AFC), anti-Mullerian hormone (AMH); (b) understand if the OSI might vary in the same patient when a different COS regimen is applied, (c) assess if the OSI could be an independent predictor of clinical pregnancy, and (d) compare the OSI with the total number of retrieved oocytes in predicting clinical pregnancy after IVF.

## 2. Methods

### 2.1. Patients

The present study included 3353 IVF cycles performed in 1612 patients, in which a COS with gonadotropins plus a GnRH-analogue was completed and the oocytes were retrieved.

Among these patients, 209 underwent two consecutive cycles in which the COS regimen was changed from a GnRH-agonist long protocol to a GnRH-antagonist protocol (or vice-versa); overall, this patients’ subgroup underwent 418 cycles, 226 with the GnRH-agonist long protocol, and 192 with the GnRH-antagonist protocol.

### 2.2. Controlled Ovarian Stimulation (COS)

The long GnRH-agonist protocol (n = 2299 cycles) was accomplished by administering 600 μg intranasal Buserelin (Suprefact, Hoechst, Germany) from the mid-luteal phase of the preceding cycle in order to obtain pituitary functional suppression. In the GnRH-antagonist protocol (n = 1054 cycles), ganirelix (Orgalutran, MSD, Readington, NJ, USA) was administered (0.25 mg/d s.c.) from stimulation day 5, according to a fixed regimen. Either the recombinant FSH (rFSH; Gonal F, Merck, Darmstadt, Germany or Puregon, MSD, Readington, NJ, USA), or the human menopausal gonadotropin (hMG; Meropur, Ferring, Germany) was administered in order to stimulate multiple follicular growth. The gonadotropin starting dose (100–375 IU) was individually chosen according to the age, BMI, circulating AMH, and antral follicle count (AFC), and was then adjusted from stimulation day 5–7 according to the ovarian response at the first checkpoint, during which the transvaginal US (TV-US) plus the serum estradiol (E2) measurement were performed. The follicular growth was then monitored by the TV-US plus serum E2 every 2–3 days, and when the leading follicle reached 17–18 mm diameter, with appropriate E2 circulating levels, ovulation was triggered by a single subcutaneous injection of 10,000 IU hCG (Gonasi HP, Ibsa, Lugano, Switzerland).

### 2.3. Oocyte Retrieval, In Vitro Culture, Embryo Transfer and Pregnancy Assessment

The oocyte pick-up (OPU) was performed approximately 36 h after the hCG injection by TV-US-guided aspiration, under a local anesthesia (paracervical block). The oocytes were immediately retrieved from the cumulus–oocyte complexes, and the mature, metaphase II (MII) oocytes were then inseminated using either IVF or ICSI, as indicated. One or two embryos were transferred in uteri after 2–5 days of in vitro culture using a soft catheter (Sydney, Cook, Australia) under TV-US guidance, as previously described by our group [13]. The intravaginal natural progesterone (Crinone 8, Merck, Germany, 180 mg/day for 15 days) was used for luteal support, starting two days after OPU. The pregnancy was assessed by a serum hCG assay 14 days after the ET and then confirmed when at least one gestational sac was visualized by the transvaginal US after two further weeks (clinical pregnancy).

### 2.4. Statistical Analysis

The following variables were registered for each patient and each cycle: age, BMI, AFC, AMH, type of COS regimen (GnRH-agonist “long” or GnRH-antagonist), the total and daily dose of gonadotropins, the number of retrieved oocytes, the number of mature (MII) oocytes, the fertilization rate, the pregnancy rate (positive hCG test)/ET and clinical pregnancy rate/ET. The Ovarian Sensitivity Index (OSI) was calculated by applying the following formula [Huber 2013]: (number of retrieved oocytes/total gonadotropin dose) × 1000.

The continuous variables were described as mean ± standard deviation (SD) or as median and interquartile range (IQR), according to their distribution; the categorical variables were reported as percentages. For the correlation analysis among the continuous variables, the Pearson’s or Spearman’s coefficients were calculated, according to the distribution. The continuous variables with a skewed distribution were submitted to a logarithmic transformation, after which they turned out to be normally distributed. The variables that turned out to be significantly related to the OSI at the univariate correlation analysis were selected as independent factors in a multivariable regression model, in which the OSI was the dependent variable. A further multivariable logistic regression analysis was performed in order to assess whether the OSI could be a predictor of clinical pregnancy independent from the age, BMI, AMH and AFC.

All analyses were performed using the SAS software 9.4 for Windows (SAS Institute, Cary, NC, USA), setting the significance threshold at *p* = 0.05.

## 3. Results

The main clinical characteristics of the patients included in the study and the outcome of their 3353 IVF cycles are reported in the Supplementary Table S1. Considering all the cycles, the mean OSI was 2.6 ± 2.2 (range 0.2–17.1). The relationship between the OSI, other clinical data (age, BMI) or biomarkers (AMH, AFC) is shown in Table 1: the OSI was significantly and inversely related to age and BMI, and significantly and directly related to the AMH and AFC. A multivariable linear regression analysis confirmed that all the variables were independently related to the OSI (Table 2).

**Figure 1:**
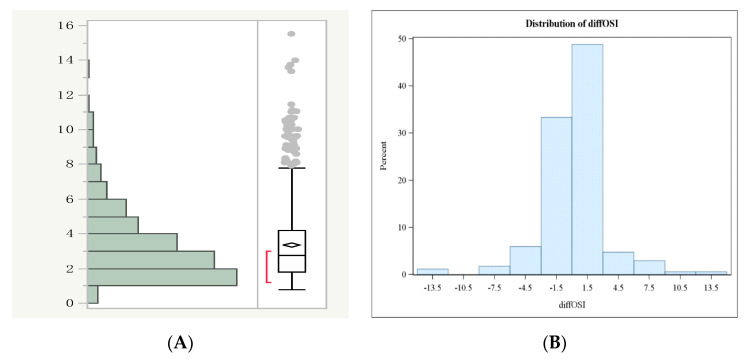
(**A**) Skewed distribution of OSI values (left) and the normal distribution after logarithmic conversion together with the diagram of quartiles (right). The cycles were subdivided into three subgroups according to the quartile of the OSI distribution: (a) the OSI < 0.17 (lower than the 25th quartile; low OSI; n = 623), (b) OSI 0.17–1.59 (between the 25th and the 75th quartile; the intermediate OSI; n = 1846), and (c) the OSI > 0.59 (higher than 75th quartile; high OSI, n = 884). (**B**) The distribution of the difference in the OSI (diffOSI) observed in patients who underwent two consecutive COS cycles changing the COS regimen from GnRH-agonist long protocol to GnRH-antagonist protocol (or vice versa). Only 18% of the patients showed a diffOSI > 1.5 between one COS protocol and the other.

The overall distribution of the OSI was clearly skewed toward lower values, but after the logarithmic transformation it became normal, following a Gaussian curve (Figure 1A, left); the cycles were then subdivided into three subgroups according to the quartile of the OSI distribution (Figure 1A, right): (a) OSI < 0.17 (lower than the 25th quartile; low OSI; n = 623), (b) OSI 0.17–1.59 (between the 25th and the 75th quartile; intermediate OSI; n = 1846), and (c) OSI > 0.59 (higher than 75th quartile; high OSI, n = 884). A high OSI quartile (>75° centile) was associated with a significantly higher pregnancy rate (PR) and the clinical pregnancy rate (CPR), whereas the mean age of this subgroup was not significantly different from that of the other two subgroups (Table 3).

Considering all the cycles, they were subdivided into three groups according to the total number of retrieved oocytes: (a) the cycles with less than 6 oocytes (poor retrieval), (b) the cycles with 6–13 oocytes (normal retrieval), and (c) the cycles with more than 13 oocytes (high retrieval). Although the subgroup of oocyte retrieval (poor, normal or high) was significantly related to the CPR/ET (*p* < 0.002), the OSI quartile showed a higher correlation with the CPR/ET (*p* < 0.0001).

In a multivariable logistic regression model, the OSI was positively correlated to clinical pregnancy independently from the age, BMI, AFC and AMH (Table 4).

Considering only the 209 patients who underwent two consecutive cycles changing the COS regimen, we found that the OSI was similar comparing 226 cycles with the GnRH-agonist and 192 cycles with the GnRH-antagonist (3.7 ± 4.2 vs. 3.9 ± 4.1, respectively, *p* = 0.44). We also analyzed the consistency of the OSI in each individual patient after the two regimens (diffOSI): the distribution of diffOSI showed that in 82% of the patients (n = 138) the OSI intra-patient variation was remarkably low (±1.5). Only 18% of the patients (n = 30) showed a diffOSI > 1.5 between one COS protocol and the other: half of them had a higher OSI with a GnRH-agonist regimen, the others with a GnRH-antagonist (Figure 1B). The remarkable inter-cycle consistency of the OSI was also confirmed by multivariable linear regression, that showed that the type of COS protocol did not significantly affect it (not shown).

## 4. Discussion

Within the concept of individualized COS (iCOS), the starting dose of gonadotropins is planned considering the clinical (age, BMI), hormonal (AMH) and US (AFC) biomarkers of ovarian responsiveness, singularly or combined in more complex models, that aim to predict how many oocytes will be retrieved [5]. Indeed, the number of oocytes retrieved at the OPU was shown to significantly affect the probability of obtaining a live birth with a fresh embryo transfer [1], as well as the cumulative live birth rate obtained after also transferring in utero all thawed embryos [2,3].

The number of retrieved oocytes, however, may be affected by a series of factors, some of which depend on the physician’s choice (e.g., the type and dose of gonadotropins), some others on the intrinsic sensitivity of the ovary to hormonal stimulation. The polymorphic variability of the FSH-receptor [7] and the individual rhythm and extent of the follicular maturation waves [8] represent examples of the complex regulation of follicular recruitment. A relevant proportion of the patients may display a markedly lower (or higher) than expected response to the COS, and despite the dose adjustments they may finally obtain a number of oocytes that is not properly reflecting the full potential of their ovary. This may be only expressed if the hormonal stimulation is performed at maximal doses, which is often impossible in clinical practice for safety reasons, such as the prevention of severe OHSS. The final result of this combination of elements is that the number of retrieved oocytes does not always reflect the true ovarian sensitivity to gonadotropins.

The Ovarian Sensitivity Index (OSI) links the number of retrieved oocytes to the degree of hormonal stimulation, expressing how many units of exogenous gonadotropins are needed to obtain each oocyte [11]. Huber et al. studied a large non-selected IVF population and showed that the OSI was normally distributed after logarithmic transformation and could be effectively used to stratify IVF patients into poor, normal, or high responders to gonadotropins [12]. These authors showed that the probability of a clinical pregnancy was directly related to the absolute number of retrieved oocytes and with the OSI; however, the pregnancy rate was comparable for the patients obtaining a high number of oocytes (16 or more) after receiving a high gonadotropin dose (low OSI), and for the patients with a significantly lower number of available oocytes (less than 6), but obtained after a low dose stimulation (high OSI) (25.4% vs. 27.2%, respectively) [12]. These results may suggest the existence of some detrimental effect of a high gonadotropin stimulus on the quality of oocytes or endometrium. They also suggest that the patients with a poorly responsive ovary, who need a high gonadotropin dose, are ab initio less prone to pregnancy, as they may be pharmacologically forced to produce more oocytes, but of poor quality.

In the present study, we aimed to better assess the characteristics of the OSI. In agreement with previous reports [11], we observed that the OSI significantly correlated with biomarkers that are currently used to predict the ovarian responsiveness, such as the age, BMI, AFC, and AMH; the correlation was direct with the AFC and AMH, whereas it was inverse with age and BMI.

Moreover, subdividing patients according to the quartile of the OSI distribution, we observed that a high OSI was associated with a significantly higher pregnancy rate (PR) and clinical pregnancy rate (CPR) independent of age, suggesting that the women who preserve a highly sensitive ovary have a good reproductive prognosis even at reproductively advanced ages. When the same kind of stratification was done according to the number of retrieved oocytes, obtaining subgroups of patients with different oocyte yield, we observed that the CPR was significantly correlated also with the total oocyte yield, but the correlation with the OSI was stronger (*p* < 0.0001 vs. 0.002, respectively). Interestingly, it was previously shown that the correlation coefficients of the OSI with age, AFC and AMH were significantly higher than those of the oocyte number itself [14]. Taken together, these observations suggest that the OSI reflects ovarian responsiveness, and to some extent the CPR, better than the number of retrieved oocytes itself. When the correlation between the OSI and CPR was adjusted by all the variables significantly related to the CPR (age, BMI, AFC and AMH), it remained significant, a further indication that the OSI represents an independent predictor of success.

Evaluating the patients submitted to repeated IVF cycles, in which two different types of the COS regimen were used (long GnRH-agonist long protocol or GnRH-antagonist protocol), we observed that the OSI was remarkably consistent in 82% of patients, varying by less than 1.5 points. In those who showed a more marked intercycle variability of the OSI (18% of patients), some had higher OSI with the GnRH-agonist protocol, some using the GnRH-antagonist, suggesting that the intrinsic variability of ovarian sensitivity, rather that the COS regimen, caused the OSI variation. This was also confirmed by a multivariable linear regression, that showed that the type of COS protocol did not significantly affect the OSI. Additionally, Huber et al. observed that the OSI was quite stable in patients undergoing two consecutive IVF cycles using the same GnRH-agonist protocol [12]; our study is the first showing that the OSI has quite a good inter-cycle consistency even if the COS protocol is changed, appearing to be a patient-related (and not protocol-related) characteristic.

Overall, the results reported herein suggest that the OSI reflects quite precisely the sensitivity of the ovary to gonadotropins, and represents a reliable marker of ovarian responsiveness that may be used to predict ovarian response and to estimate the probability of clinical pregnancy even for patients who will undergo a different COS regimen. The fairly good consistency of the OSI suggests that it might be introduced into the logarithms used to calculate the ideal dose of gonadotropins for COS, possibly increasing their accuracy. Indeed, a recently published prediction model for IVF outcome, which also has a high level of accuracy in predicting clinical pregnancy, was based on a complex equation that included an evidence-based embryo score [15] and several patient- and cycle-related variables, among which the OSI appeared as one of the most relevant [16].

In conclusion, the present study shows that the OSI is significantly related to the main biomarkers of the ovarian reserve; it is an independent predictor of clinical pregnancy; it is more significantly correlated with clinical pregnancy than the number of retrieved oocytes, and is highly consistent even when the COS protocol is changed. These characteristics make the OSI quite suitable to be incorporated into more complex prediction models of IVF outcome.

## Figures and Tables

**Table 1:** Correlation among the Ovarian Sensitivity Index (OSI), clinical (age, BMI), endocrine anti-Mullerian hormone (AMH) and US antral follicle count (AFC) variables related to the ovarian responsiveness (univariate analysis). Age and BMI turned out to be negatively related to the OSI, whereas the AMH and AFC were positively related. All the correlations were statistically significant.

	Coefficient	*p*
Age (years)	−0.45426	<0.0001
BMI (kg/m*^2^*)	−0.11756	0.0188
AFC	0.50336	<0.0001
AMH (ng/mL)	0.45017	<0.0001

**Table 2:** Correlation among the OSI, clinical (age, BMI), endocrine (AMH) and US (AFC) variables related to ovarian responsiveness (multivariable linear regression analysis). Age and BMI turned out to be negatively related with the OSI, whereas the AMH and AFC were positively related. All the correlations were statistically significant.

	Coefficient	*p*
Age (years)	−0.00041	0.0004
BMI (kg/m^2^)	−0.26657	<0001
AFC	0.11026	<0001
AMH (ng/mL)	0.18341	0.0021

**Table 3:** Relationship between the OSI, pregnancy rate (PR/ET), and clinical pregnancy rate (CPR/ET). The PR/ET and CPR/ET were significantly higher (*p* < 0.0001) in cycles with a high OSI (>75° quartile) than in cycles with a low (<25° quartile) or an intermediate (25°–75° quartile) OSI, despite the comparable mean patients’ age. * *p* < 0.0001 vs. other quartiles.

	PR/ET (%)	CPR/ET (%)	Age (years)
OSI < 2 5° quartile (low OSI; n = 423)	26	18.6	40.8
OSI 25°–75° quartile (intermediate OSI; n = 1646)	37	30.9	39.1
OSI > 75° quartile (high OSI; n = 676)	54 *	46.5 *	38.2

**Table 4:** Multivariable analysis of the variables associated with clinical pregnancy (CPR/ET). The results are presented with an odds ratio (OR) and 95% confidence interval (95% CI) estimates.

	OR	95% Wald Confidence Limits	
Age	0.943	0.913	0.975
BMI	0.973	0.935	1.012
AMH	0.940	0.890	0.993
AFC	1.029	1.011	1.046
OSI	1.078	1.035	1.121

## Supplementary Materials

The following are available online at https://www.mdpi.com/2077-0383/9/6/1914/s1, Table S1: Main clinical characteristics of the patients and of their 3,353 IVF cycles, expressed as mean ± SD (range) or percentage.
