# Supplementary material for: The Ovarian Sensitivity Index (OSI) Significantly Correlates with Ovarian Reserve Biomarkers, Is More Predictive of Clinical Pregnancy than the Total Number of Oocytes, and Is Consistent in Consecutive IVF Cycles
Source: J Clin Med. 2020 Jun 18;9(6):1914. doi: 10.3390/jcm9061914 (PMC7355532; doi:10.3390/jcm9061914)
Supplement: Supplementary file 1 [file jcm-09-01914-s001.pdf]

Supplementary Table 1. Main clinical characteristics of the patients and of their 3,353 IVF cycles, expressed as mean±SD (range) or percentage.

**Age (years)**	**38,3 ± 4,3 (19-43)**
BMI (kg/m^2)	21,6 ± 3,2 (15-40)
Antral Follicle Count (AFC)	15,1 ± 10,8 (3-38)
AMH (ng/ml)	2,3 ± 2.2 (0.1-12.9)
Total dose of gonadotropins (IU)	2734 ± 1356 (600-9000)
Daily gonadotropin dose (IU)	263 ± 96 (75-450)
N. of retrieved oocytes	5,9 ± 4,8 (1-31)
N. of mature (MII) oocytes	5.2 ± 3.4 (1-26)
Fertilization rate (%)	68.5
Positive hCG test/ET (%)	40,3
Clinical (US) Pregnancy Rate/ET (%)	33,7
